# Budd-Chiari syndrome secondary to retro-hepatic vena cava web: An unusual cause of epigastric pain

**DOI:** 10.1016/j.radcr.2022.12.044

**Published:** 2023-01-07

**Authors:** Manar Ezzahi, Ennasery Zaid, Aassouani Farid, Soukayna Allali, Nizar El Bouardi, Meriem Haloua, Abid Hakima, Badreeddine Alami, Meriem Boubbou, Mustapha Maaroufi, Moulay Youssef Alaoui Lamrani

**Affiliations:** a Department of Radiology and Interventional Imaging, CHU Hassan II Fez, Sidi Mohammed Ben Abdellah University, Centre Hospitalier Hrazem, BP:1835 Atlas, Fès, Avenue Hassan II, Fez 30050, Morocco; b Department of Gastro-Hepato-Enterology - CHU Hassan II Fez, Sidi Mohammed Ben Abdellah University, Fez, Morocco

**Keywords:** Vena cava, Supra-hepatic veins, Web, MOIVC, Budd-Chiari, Ultrasound, CT, MOIVC, membranous obstruction of inferior vena cava, BCS, Budd-Chiari syndrome, IVC, inferior vena cava, CT, computed tomography, MRI, magnetic resonance imaging, WCC, white count cell, HBs Ag, hepatitis B surface antigen, HBc, hepatitis B core, HCV, hepatitis C virus, ALP, alkaline phosphatase, ALT, alanine transaminase, AST, aspartate aminotransferase, GGT, gamma-glutamyl transferase, Anti-LKM, anti-liver and kidney microsome, Anti-SLA, anti-soluble liver antigen, PT, prothrombin time, MPS, myeloproliferative syndromes, APS, antiphospholipid syndrome, TIPS, trans-jugular intra-hepatic portosystemic shunt

## Abstract

Epigastric abdominal pain is a common indication for consultation. In the majority of cases, medical history, clinical examination and routine biological exams allow for an easy diagnosis. Sometimes the symptomatology is unusual, in which case it is essential to perform a complete clinical examination and to use various imaging techniques to search for eventual atypical causes. Membranous obstruction of inferior vena cava is a rare cause of such a phenomenon. We describe a Budd-Chiari syndrome caused by membranous obstruction of inferior vena cava in a 66-year-old woman with no medical history as a rare cause of epigastric abdominal pain. We will describe this clinical experience in the light of the literature and point out the contribution of radiological imaging in the diagnosis of this rare pathology.

## Introduction

Epigastric pain is a common symptom of abdominal or extra-abdominal pathology, with acute or chronic onset, requiring either surgical or medical treatment.

It requires a careful questioning and complete physical examination which will guide the request for complementary examinations.

The Budd-Chiari syndrome (BCS) is an uncommon cause of such pain, characterized by hepatic venous outflow obstruction above the hepatic venules regardless of etiology, position, or severity of the obstruction [1].

It is classified as primary or secondary depending on its pathologic features. The primary type is due to endoluminal venous obstruction by thrombosis or a fibrous membrane “Web,” revealing an underlying pro-thrombotic state [2]. The secondary type is defined by an obstruction of the same anatomic structures by material of extravascular origin [3].

In the primary BCS, the origin of the fibrous membrane (web) was initially considered to be congenital but recent reports suggest that the vena cava web corresponds to post-thrombotic sequelae with the regressed thrombus replaced by a fibrous laminar structure which can lead to hepatic outflow obstruction.

Imaging diagnosis is mainly based on Doppler ultrasound; computed tomography (CT) scan is usually of little interest in this pathology. It finds its place in BCS secondary to hepatic or retroperitoneal tumors. Magnetic resonance imaging (MRI) is indicated in severe and chronic forms where the clinical picture is ambiguous [3].

Literature search reveals that membranous obstruction of the inferior vena cava is a common cause of BCS in Asia and South Africa but has rarely been reported in Caucasian populations [4,5].

In this case report, we present the diagnosis and management of BCS secondary to retro-hepatic vena cava web revealed by a subacute onset of atypical epigastric pain in a Caucasian patient, in whom the diagnosis was confirmed by ultrasound imaging.

## Case Presentation

The patient, L.F, is a 66-year-old Caucasian housewife who was referred to the gastro-hepato-enterology department of the university hospital center Hassan II of Fez for exploration of an atypical epigastric pain. The patient is without any pathological medical or surgical history.

She reports a subacute onset of continuous epigastric pain over the 3 previous weeks. This pain was extending to the right hypochondriac region, and not responding to symptomatic treatment. She didn't report any episodes of vomiting or signs of upper or lower digestive hemorrhage.

The first physical examination revealed a well orientated patient, with neither asterixis, jaundice, or fever. Hemodynamic and respiratory values were normal, with 115/75 mmHg of blood pressure, a 98/min heart rate and a 21 cycles/min respiratory rate. The nutritional status was a bit impaired, with a body mass index of 19.5 kg/m^2^.

The abdominal examination revealed epigastric tenderness, with firm hepatomegaly (liver edge was palpable 2.5 cm below the right costal margin) and presence of ascites, with edema of the lower extremities up to the ankles, there was no evidence of splenomegaly, dilated abdominal wall veins or spider angioma.

Initial laboratory tests were as follows:

The complete blood count cell showed white count cell (WCC) of 6598 × 10^9^/L (reference range: 4.5-11.00 × 10^9^/L), hemoglobin level of 12 g/dL (reference range: 10-15.5 g/dL), and platelet count of 355 × 10^9^/dL (reference range: 150-400.10^9^ g/dL). C-reactive protein (CRP) was at 30 mg/L (reference range < 4).

Liver function tests showed;

• Albumin: 18 g/L (35-52).
• ALP (alkaline phosphatase): 221 units (30-120 UI/L)
• ALT (alanine transaminase): 29 units (0-35 UI/L)
• AST (aspartate aminotransferase): 44 units (0-35 UI/L)
• GGT (Gamma-glutamyl transferase): 187 units (0-38 UI/L)
• Total bilirubin: 44 mg/L (3-12 mg/L)
• Direct bilirubin: 28mg/L (0-2 mg/L)
• PT (Prothrombin time): 55%.

Lipid tests, urea, electrolytes and creatinine were all in the normal values.

Serology and immunological blood tests showed negative hepatitis B surface antigen (HBs Ag), positive anti-hepatitis B core (HBc) antibody, negative anti-hepatitis C virus (HCV) antibody.

Serum protein electrophoresis showed no hyper-gamma-globulinemia; negative antinuclear antibodies, negative anti-liver and kidney microsome (anti-LKM), negative anti-soluble liver antigen (anti-SLA), negative anti smooth muscle antibodies, negative anti-mitochondria antibodies, and negative anti-cytosol antibodies.

**Fig. 1:**
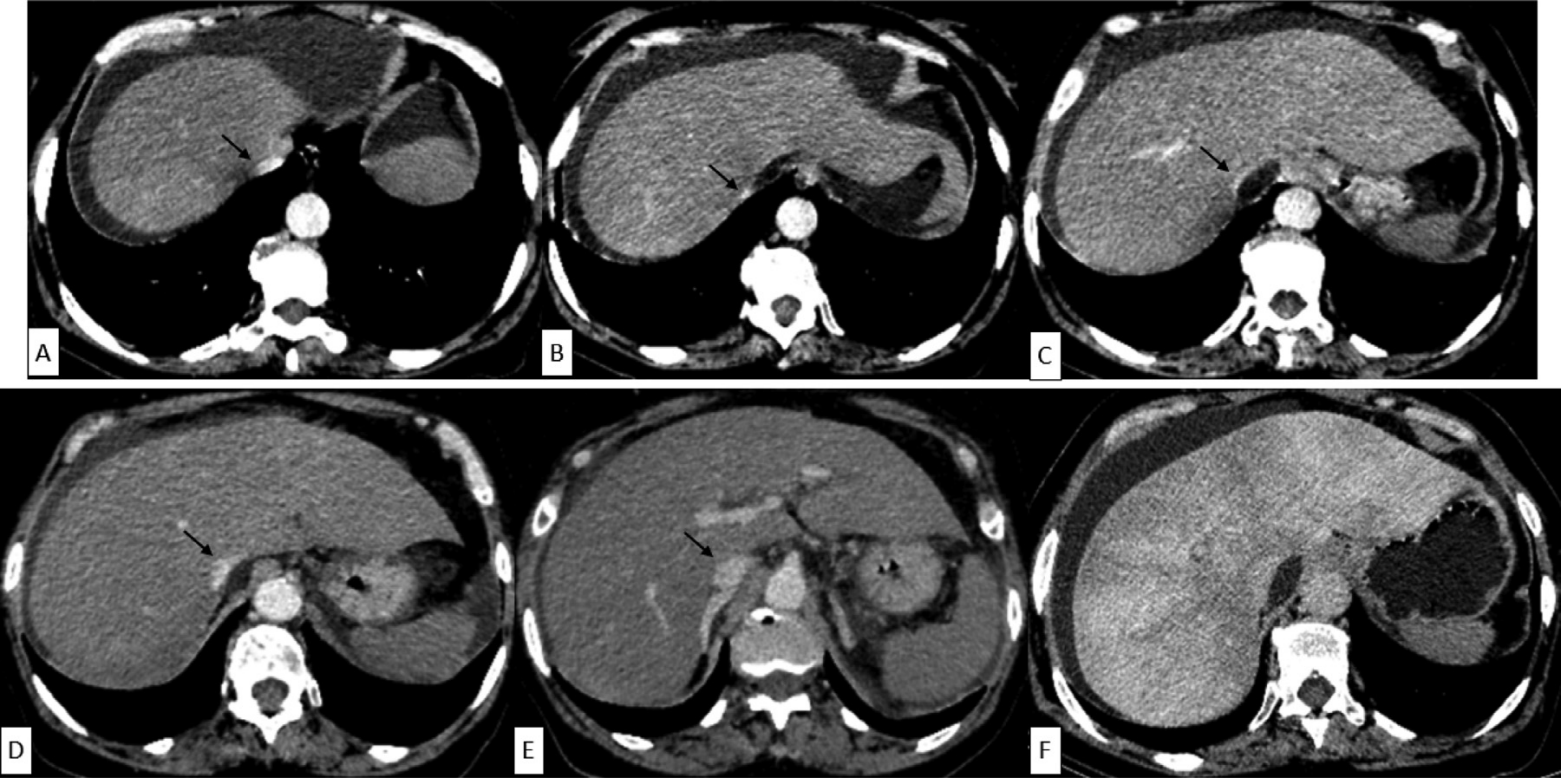
(A-F) Abdominal CT scan showing hypertrophy of right liver (C-F) with heterogeneous liver enhancement at the portal and delayed phases (F), without visualization of supra-hepatic main veins and retracted appearance of retro-hepatic vena cava (black arrows) which still minimally opacified at the periphery (B,C). The supra-hepatic and the sub-hepatic inferior vena cava were well opacified (A,E).

Initially, an abdominal CT angiography was realized and showed:

A discrete hepatomegaly with regular and smooth contours in non-enhanced CT, with hypertrophy of the right liver. The parenchymography study at the arterial, portal, and delated phases after contrast injection showed heterogeneous liver enhancement at the portal and delayed phases (Fig. 1F), without depicting of any focal hepatic lesion. The study of the hepatic vascular structures revealed the absence of visualization of supra-hepatic main veins with a retracted appearance of retro-hepatic vena cava which still minimally opacified at the periphery (Figs. 1B and C), with small retro-caval fluid collection, and peritoneal diffuse ascites.

The supra-hepatic and the sub-hepatic inferior vena cava were well opacified (Figs. 1A and E).

The portal vein was of normal size. The study didn't objective any signs of chronicity of BCS, in particular caudate lobe atrophy, hypotrophy of peripheral liver, or collateral channels.

In order to complete the study of supra-hepatic veins, and inferior vena cava, an abdominal ultrasound was performed.

**Fig. 2:**
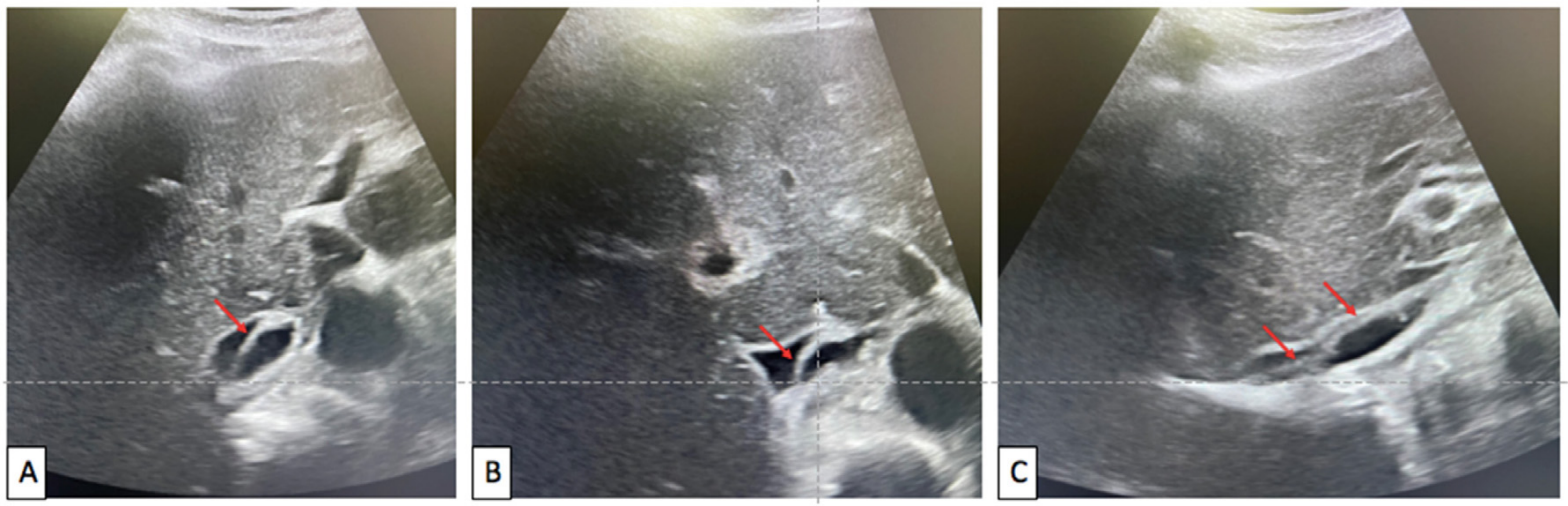
Vena cava ultrasound in B-mode in axial (A,B) and sagittal (C) view: Red arrows point the endoluminal web inside the retro-hepatic vena cava.

The Mode-B study of retro-hepatic inferior vena cava objectified (Videos 1 and 2): an endoluminal echogenic band (Fig. 2), floating into the lumen of the retro-hepatic inferior vena cava, mobile with respiration, not extending to hepatic veins which are permeable, with slow blood flow upstream in the sub-hepatic inferior vena cava below the level of the band.

The Doppler study objectified (Video 3): opacification of the lumen of vena cava, with no opacification of the part of the IVC (inferior vena cava) behind the endoluminal web. It shows also a good opacification of supra-hepatic veins with no reversed hepatic flow.

Based on these elements, we made the diagnosis of subacute BCS.

A complementary abdominal MRI was performed, but didn't show the endoluminal web or bring any additional findings.

The decision of the treating physician afterwards was to put the patient on antidiuretics with albumin infusion and anti-vitamin K therapy.

Thereafter the patient showed positive clinical and biological improvement after fluid and effective anticoagulation therapy then was discharged from the hospital.

## Discussion

BCS was first described as an obliterative endophlebitis of the hepatic veins. Currently, it involves a wide variety of disorders, all involving obstruction of hepatic venous outflow above the hepatic venules, regardless of the etiology, position, or severity of the obstruction [1].

BCS should always be suspected in the presence of these findings: the triad associating hepatomegaly, ascites, and upper abdominal pain, the presence of fulminant liver failure with hepatomegaly and ascites; a chronic liver disease of unexplained etiology; or a liver disease in a patient with pro-thrombotic state.

The disturbances of the biological assessment are essentially related to the speed and extent of venous obstruction. In acute forms hepatocellular insufficiency can be severe, rarely fulminant or sub-lethal with high transaminases, greater than 5 times, a prothrombin level <50%, a protid-rich ascites and renal insufficiency. In chronic forms, cytolysis is often less than 5 times normal, prothrombin level >50%, hypoalbuminemia and renal failure are common [3].

BCS is classified as primary or secondary depending on its pathologic features.

The primary type is due to endoluminal venous obstruction by thrombosis or its fibrous sequel, revealing an underlying pro-thrombotic state. The secondary type is defined by an obstruction of the same anatomic structures by material of extravascular origin [2].

Membranous obstruction of the cava vein (primary BCS) was first described by Thompson and Turnbull in 1912; it may be isolated or associated with obstruction of the supraspinal veins [4], [5], [6].

There has been much debate in the literature about the etiology of primary BCS.

The origin of the inferior vena cava web or membrane was ambiguous: it was initially assumed to be a congenital disease, but certain data do not support this hypothesis due to many arguments: the age of the clinical presentation (3rd and 4th decade), there are no reports of membranous obstruction of inferior vena cava (MOIVC) in fetuses, neonates, or infants, and reports documenting the transformation and organization of a thrombosis into fibrotic and elastic bands [5], [6], [7].

In patients with BCS, thrombosis is associated in 2/3 of cases with one or more prothrombotic conditions, the most frequent are: myeloproliferative syndromes (MPS), antiphospholipid syndrome (APS), paroxysmal nocturnal hemoglobinuria, and hereditary thrombogenic etiologies. The ethiopathological mechanism of these conditions remains ambiguous to this day but the frequent association has supported this view [8,9].

Imaging diagnosis is mainly based on [10,11]:

Doppler ultrasound: obstruction of the main hepatic veins can be recognized by Doppler ultrasound alone in more than 80% of cases. Older thromboses appear as a thin hyperechogenic cord on the path of the corresponding vein. Recent thrombosis presents as an enlarged vein filled with hypoechogenic material. Pulsed and color Doppler ultrasound shows a continuous velocity modulated flow in the vein upstream of the stenosis instead of the usual tri-phasic morphology. In color Doppler, venous return bypass routes can be visualized.

CT: It is of little value in this condition. The stenotic lesions of the supra hepatic veins are poorly visible, even if thrombosis can be demonstrated. Only indirect signs such as dysmorphia, ascites, and a hepatic parenchyma heterogeneity after injection of contrast point to this diagnosis. It finds its place especially in BCS secondary to extrinsic compression or invasion of inferior vena cava or hepatic veins by hepatic or retroperitoneal tumors.

MRI: It is indicated in severe forms, where an emergency hepatic transplant may be discussed, and in chronic forms where the clinical picture is ambiguous. A double semiology is found, that of the hepatic parenchyma and that of the vessels:

Ø Parenchymal abnormalities: a hypersignal on T2-weighted sequences is found on the affected segments in the acute phase, it is secondary to the congestion of the acute phase. In chronic phases, on the contrary, the signal of the liver decreases compared to that of the spleen or segment I.
Ø Vascular abnormalities: the semiology of vascular thrombosis is found; high signal on spin echo sequences and no signal on gradient echo.

In our patient, the abdominal MRI showed heterogeneous liver, but didn't help in the vascular study since the web (or membrane) of vena cava was not depicted.

In addition, magnetic resonance imaging may be also used to differentiate slow-flowing blood from intraluminal thrombus by examining first- and second-echo images; slow flow usually induces an increase in relative signal density on a second-echo image, while thrombus exhibits either a lesser increase or a decrease in relative signal intensity (paradoxical enhancement) [12].

Treatment of BCS is the most challenging subject, as therapeutical choices depend on a multitude of variables (anatomical conditions, underlying disease, and liver function) [1]. In Europe, the therapeutic recommendations are organized in an algorithm, first a medical treatment based on anticoagulants is instituted in all patients, if unsuccessful an endovascular re-permeabilization by angioplasty, stent or thrombolysis is performed. In case of persistence or aggravation of symptoms, a porto-systemic derivation by trans-jugular intra-hepatic portosystemic shunt (TIPS) is then considered, and finally hepatic transplantation is proposed as the last therapeutic alternative [13].

## Summary

MOIVC or inferior vena cava web is a rare vascular pathology that should be considered in a patient presenting with unusual abdominal pain.

Recent studies suggest that vena cava webs most likely occur as result of an organized thrombus. The etiologies are the same of thrombosis, dominated by myeloproliferative syndromes.

Diagnosis is based primarily on Doppler ultrasound, which should be used as the initial complementary procedure.

The study of the supra hepatic veins and inferior vena cava should be systematic during any abdominal ultrasound scan, especially in patients presenting with diffuse abdominal pain or ascites of undetermined origin.

The discovery of this disorder should prompt an exhaustive etiologic investigation in search of a potentially curable adjacent pathology.

## Ethics approval and consent to participate

Not applicable.

## Availability of data and materials

The data sets are generated on the data system of the CHU Hassan II of Fes, including the biological data and the interventional report.

## Patient consent

I, the author of the article: Budd-Chiari syndrome secondary to retro-hepatic vena cava web: an unusual cause of epigastric pain, approve that the patient gives her consent for information to be published in *Radiology Case Reports*.

## Appendix Supplementary materials

Image, application 1
