# Supplementary material for: Budd-Chiari syndrome secondary to retro-hepatic vena cava web: An unusual cause of epigastric pain
Source: Radiol Case Rep. 2023 Jan 7;18(3):1088–92. doi: 10.1016/j.radcr.2022.12.044 (PMC9849858; doi:10.1016/j.radcr.2022.12.044)
Supplement: Supplementary file 1 [file mmc1.docx]

**Supplemental Material (3 videos)**

**
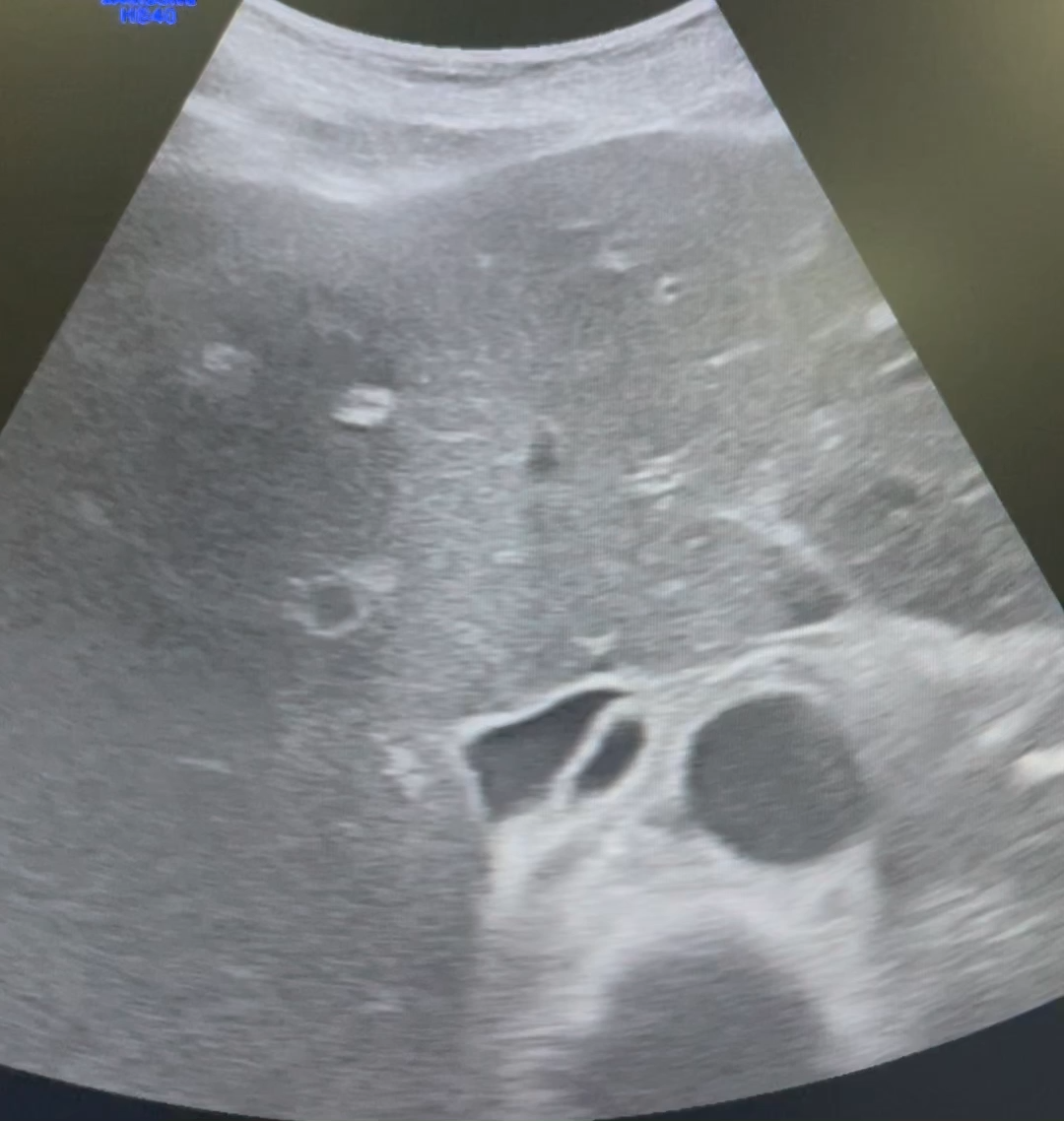
**

Video N° 1: Sagittal and axial ultrasound study (B-mode)


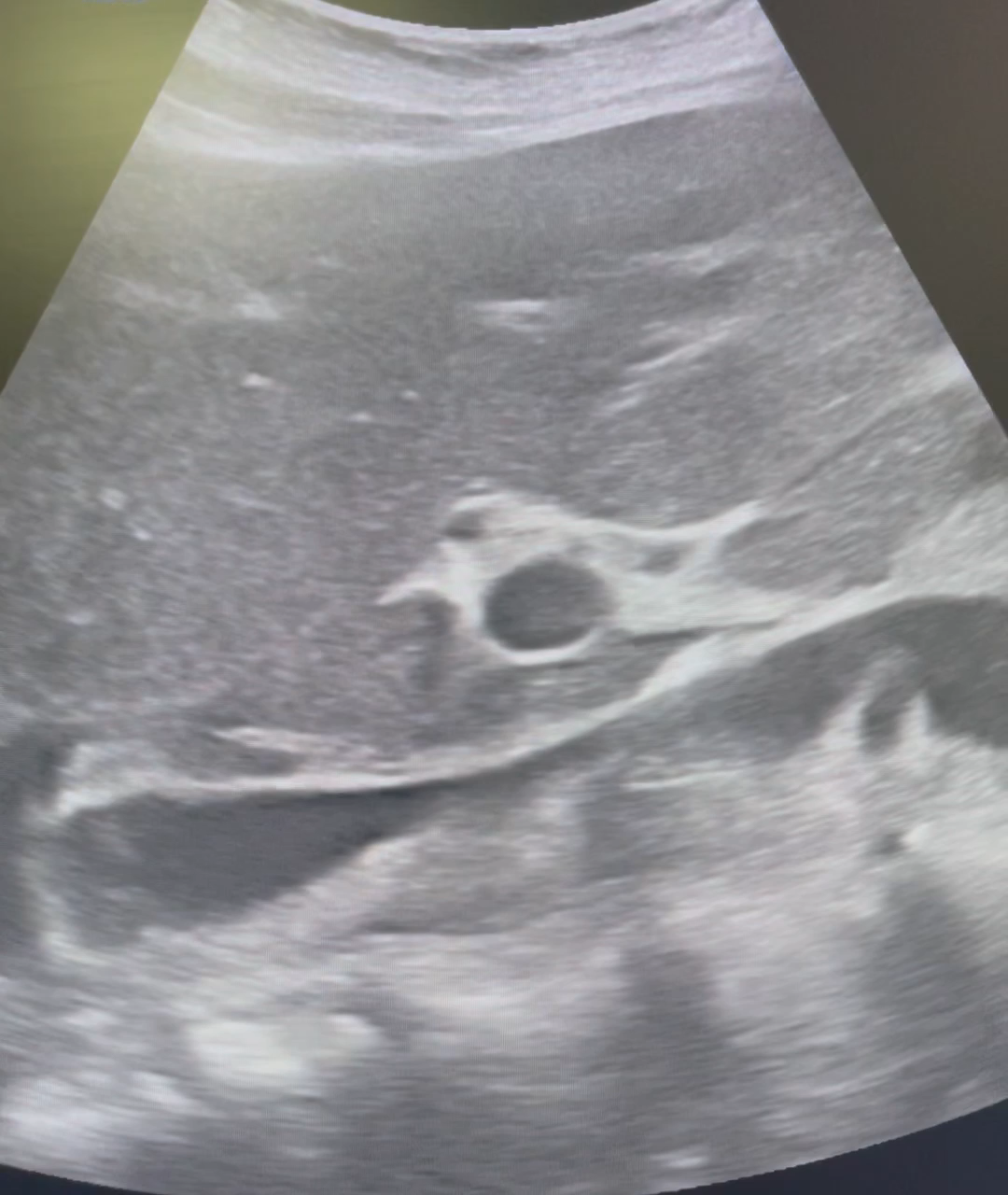


Video N° 2: Sagittal ultrasound study (B-mode)


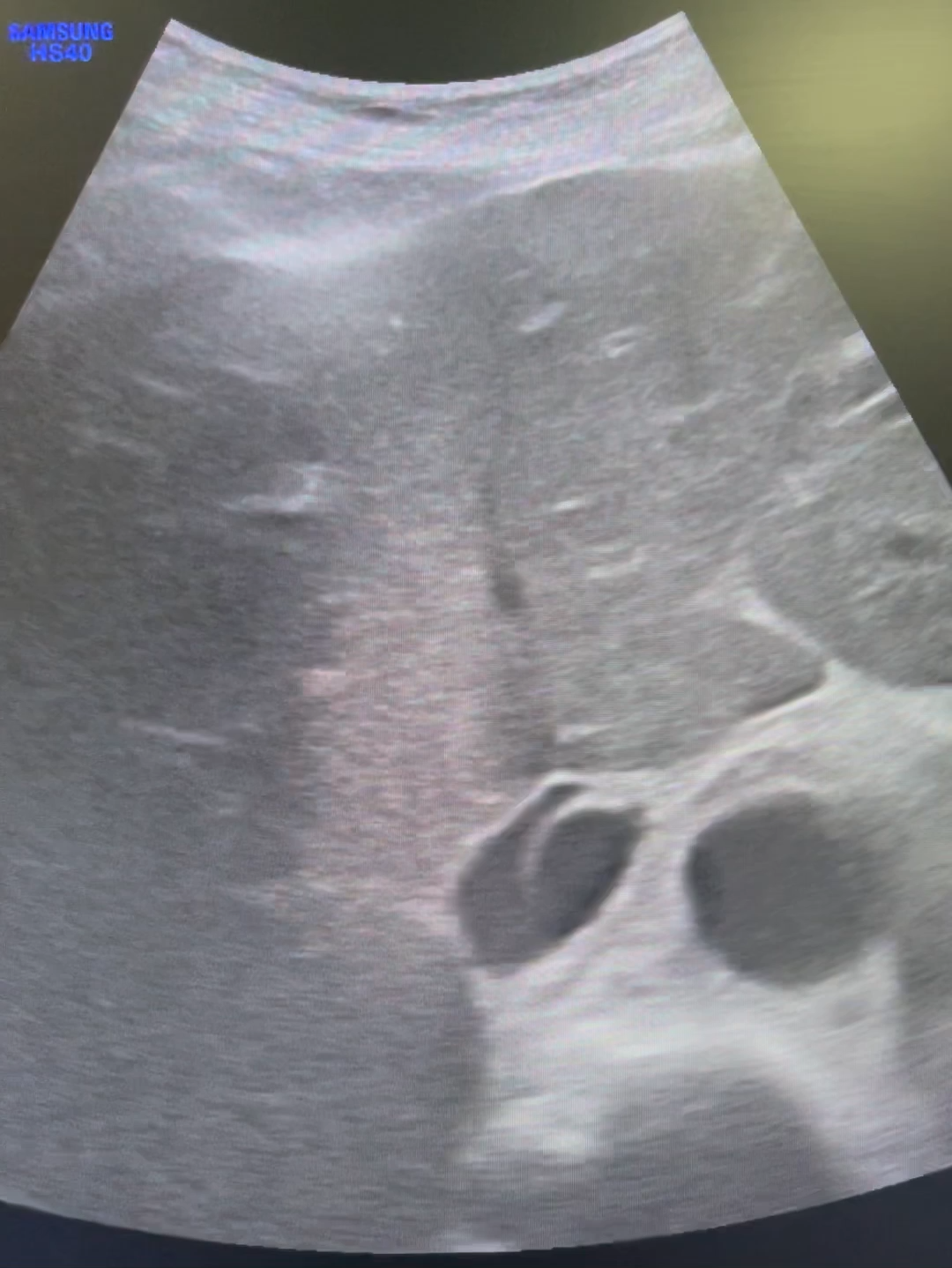


Video N° 3: Doppler ultrasound study
